# Epididymo-Orchitis Secondary to Aeromonas hydrophila: A Rare Presentation and Case Report

**DOI:** 10.7759/cureus.74326

**Published:** 2024-11-23

**Authors:** Ibrahim Bahabri, Ahmad A Hazzazi, Abdulrahman M Aldhilan, Henry Baffoe-Bonnie

**Affiliations:** 1 Department of Medicine, King Abdulaziz Medical City, Riyadh, SAU; 2 Department of Infection Prevention and Control, King Abdulaziz Medical City, Riyadh, SAU

**Keywords:** aeromonas hydrophila, catheter-related urinary tract infection, epididymo-orchitis, neurogenic bladder dysfunction, recurrent uti

## Abstract

*Aeromonas* is a group of bacteria commonly found in water sources. These bacteria are known to cause gastrointestinal and skin infections, while their association with urinary tract infections is relatively rare. Here, we present a case of *Aeromonas* epididymitis in a patient with a chronic neurogenic bladder managed with clean intermittent catheterization. This case highlights the significance of considering *Aeromonas hydrophila* as a potential pathogen in urogenital infections, especially among patients with risk factors such as catheterization and neurogenic bladder. Future research should explore the risk factors and mechanisms associated with *Aeromonas* infections in urogenital contexts and their implications for management in patients with neurogenic bladder.

## Introduction

*Aeromonas* is a genus of non-spore-forming, facultatively anaerobic, rod-shaped gram-negative bacteria [1]. They are widely distributed in various aquatic environments, including freshwater lakes, seawater, drinking water, groundwater, and wastewater [2]. Mesophilic *Aeromonas* species (typified by *A.* *hydrophila*) commonly present as self-limited diarrhea, which can progress to a chronic state if left untreated or develop into disseminated bacteremia in immunocompromised individuals, such as patients with hepatic dysfunction and hematologic malignancies [3]. Another common disease pattern involves wound infections, often resulting from exposure to contaminated water. These infections can rapidly evolve into necrotizing fasciitis, necessitating extensive surgical intervention, including amputations in severe cases [4]. Beyond the well-established gastrointestinal and wound infections, a few case reports have documented various presentations of *Aeromonas* species involving other organs. These include, but are not limited to, peritonitis in patients with cirrhosis or those undergoing peritoneal dialysis [5-6], pneumonia following near-drowning incidents [7-8], urinary tract infections (UTI) [9-10], and even prostatitis and secondary septicemia caused by *A. sobria* [11]. Genital infections caused by *Aeromonas* species are rare. Therefore, we aim to further explore the significance and range of urogenital diseases associated with *Aeromonas *by reporting a case of *Aeromonas* epididymitis in a male undergoing continuous intermittent catheterization (CIC) for chronic neurogenic bladder.

## Case presentation

A 63-year-old male with a medical history significant for type 2 diabetes, hypertension, chronic kidney disease stage 5, and a past brucellosis infection 40 years ago, as well as a neurogenic bladder status post-botox injection on clean intermittent catheterization, presented to King Abdulaziz Medical City in Riyadh, Saudi Arabia, with a chief complaint of severe, intermittent left testicular pain persisting for 10 days, unresponsive to acetaminophen. The patient reported no associated fever, dysuria, hematuria, or other complaints. Two similar episodes occurred earlier in the year, with the first occurring four months prior to this presentation, revealing a right epididymal non-drainable abscess that responded well to a two-week course of ciprofloxacin. The second episode, which occurred two months previously, involved left epididymo-orchitis with a small non-drainable epididymal tail collection and a urine culture that grew >100,000 cfu/ml of *Streptococcus agalactiae* (Group B), which responded favorably to a two-week course of ciprofloxacin. The patient denied any recent risky behaviors, extra-marital or new sexual contacts, alcohol or illicit drug intake, or exposure to water bodies.

**Figure 1:**
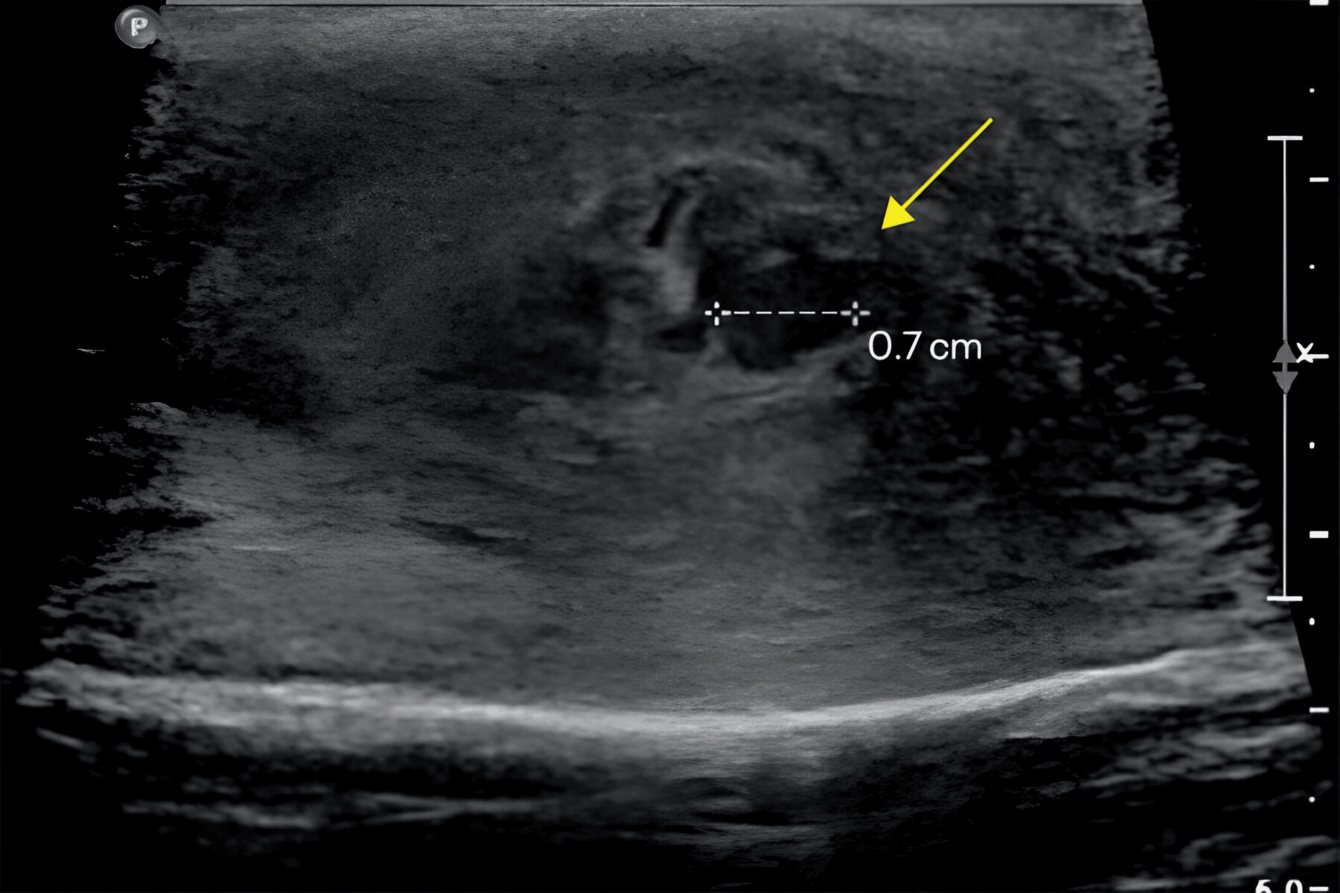
Ultrasonographic Imaging of left epididymis showing left epididymal tail collection (yellow arrow) measuring 0.7 cm with diffuse heterogeneous enlargement of the epididymal tail consistent with epididymo-orchitis.

**Figure 2:**
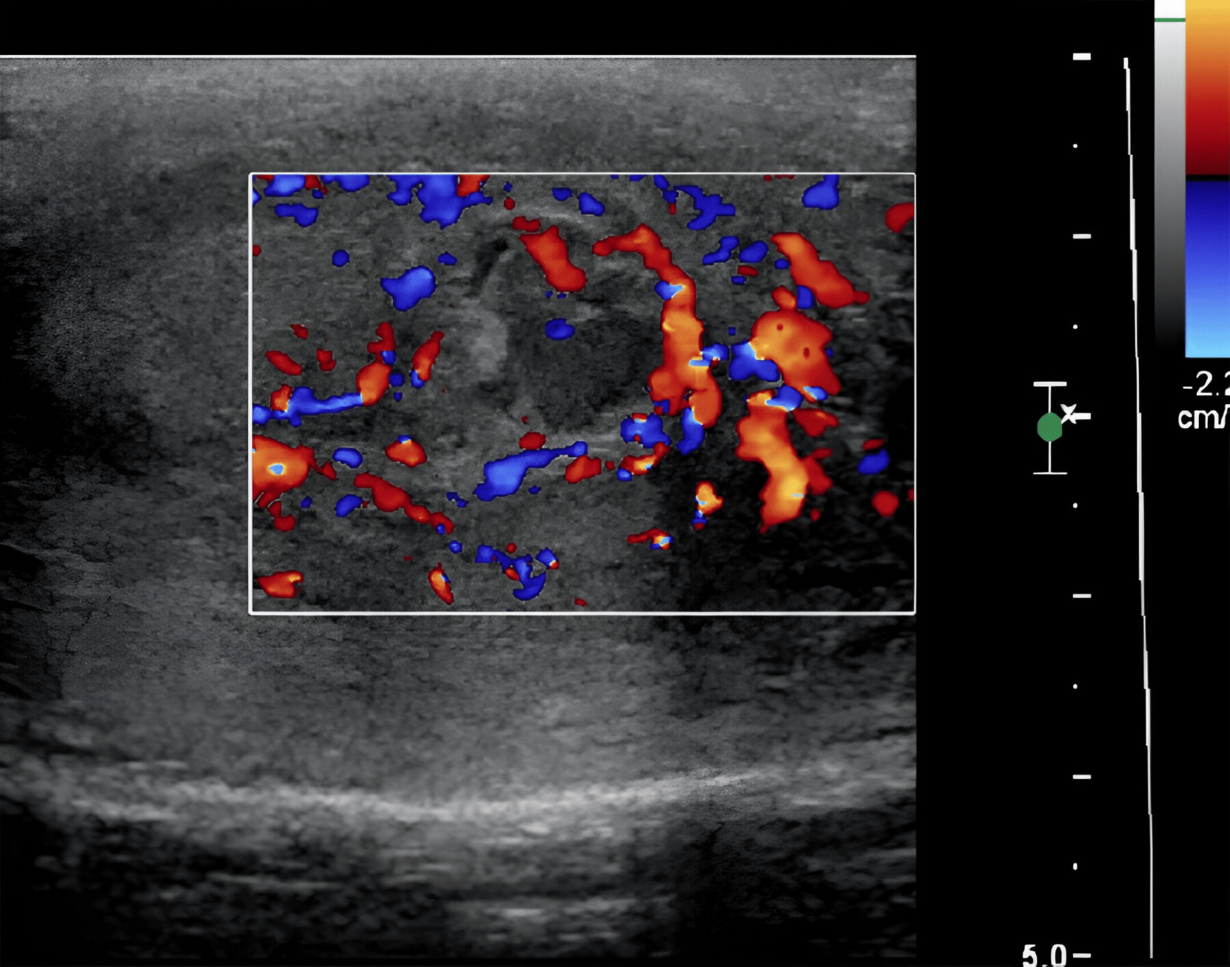
Ultrasonographic Doppler imaging of left epididymis showing increased vascularity and heterogeneous echogenicity in the left epididymal tail (white square) consistent with epididymo-orchitis.

On examination, the patient was afebrile with normal vital signs, and the abdominal examination was unremarkable. A significant swelling in the left testicular area with mild tenderness was noted. Ultrasonographic imaging of the swelling demonstrated interval improvement of the left epididymal tail collection from 2.3 cm to 0.7 cm with diffuse heterogeneous enlargement of the epididymal tail, indicating persistent left epididymo-orchitis (Figures 1, 2).

The laboratory results shown in Table 1 revealed anemia, a normal white-blood-cell count, CKD with high serum creatinine similar to his recent elevated baseline, high blood urea nitrogen, normal liver function tests, normal electrolytes apart from mild hypocalcemia, and elevated inflammatory markers. Urinalysis showed cloudy urine with elevated WBCs and leukoesterase. Urine red blood cells and nitrate were negative.

**Table 1:** Laboratory investigation results Hgb: hemoglobin, WBCs: white blood cells, BUN: blood urea nitrogen, ESR: erythrocyte sedimentation rate, CRP: C-reactive protein, PCT: procalcitonin, RBCs: red blood cells

Test	Observed value	Reference range
Hgb	99 gm/L	135-180 gm/L
WBCs	10.1 × 10^9/liter	4-11 x 10⁹g/L
Serum creatinine	588 μmol/L	62-115 umol/L
BUN	34.2 mmol/L	3-9.2 mmol/L
Calcium	2.05 mmol/L	2.2-2.5 mmol/L
ESR	110 mm/hr	0-15 mm/hr
CRP	61 mg/L	0-5 mg/L
PCT	0.37 ng/mL	0-0.05 ng/mL
Urine WBCs	317/HPF	0-5 /HPF
Urine RBCs	4/HPF	0-5/ HPF
Leukoesterase	500 leu/μL	Negative
Nitrate	Negative	Negative

Urine culture identified the growth of 90,000 cfu/ml *Aeromonas* *hydrophilia*. Susceptibility testing showed that the isolate was susceptible to meropenem, ciprofloxacin, co-trimoxazole, gentamicin, amikacin, cefepime, and cefuroxime, but resistant to piperacillin and tazobactam. The patient was treated with two weeks of meropenem followed by four weeks of ciprofloxacin, resulting in a significant improvement in symptoms upon follow-up.

## Discussion

The *Aeromonas* species primarily associated with human infections include *A. hydrophila*, *A*. *caviae*, and *A*. *veronii* *biotype* *sobria* [12]. While *Aeromonas *species are commonly linked to gastrointestinal and necrotizing skin and soft tissue infections, their role in urogenital infections, particularly genital involvement, remains uncommon [9-10]. Aeromonas UTIs in adult males have been reported and are occasionally linked to occupational exposures to aquatic environments [13-14]. In this case, the patient's CIC practice of using tap water possibly led to the direct introduction of this organism into his urogenital tract, because *Aeromonas *species are known to reside in different aquatic environments [2-4]. Previous studies have documented similar associations between *Aeromonas *and urinary infections, especially in patients with underlying conditions that cause urinary stasis or require catheter use. Neurogenic bladder, intermittent catheterization, and the presence of an indwelling device have been reported to be associated with *Aeromonas *UTIs [9-15]. Treatment with meropenem followed by ciprofloxacin proved effective in this case, highlighting the importance of antimicrobial susceptibility testing because of the variable resistance patterns exhibited by *Aeromonas *species [2]. Antibiotic selection should be guided by susceptibility profiles to ensure effective management, especially in complicated infection cases.

## Conclusions

This case highlights the significance of considering *A. hydrophila* as a potential pathogen in urogenital infections, especially among patients with risk factors such as catheterization and neurogenic bladder. The patients' assessments should include an evaluation of exposure to aquatic environments as a potential risk factor. Increased awareness and timely intervention can lead to better outcomes and prevent serious complications. Further research is needed to elucidate the mechanisms by which *Aeromonas *establishes infections in the urogenital tract and to develop guidelines for managing this rare but significant infection.
